# Florida State Medical Association

**Published:** 1874-02

**Authors:** 


Florida State Medical Association.—We present elsewhere in this number the proceedings of a convention of medical men, and of delegates from a number of local medical societies of the State of Florida, giving the organization of a State Medical Association. The jurisdiction of this young Association is a field of peculiar interest to the profession, as it is to the people, and the scientific as well as the health-seeking world, demand its cultivation. The semi-tropical character of its climate and productions, the even temperature of the atmosphere that plays over its sea-beaten shores, has turned thitherward, with hope, the health-seeking invalids of the higher or more changeable altitudes, and of the more bleak and uncongenial latitudes of the far off north and north-west. The profession, as well as the people, look with anxiety to our professional brethren of this "land of flowers" for that medical history of their State which must form the basis of the only truly scientific chart to guide the health-seeking traveler. Our brethren there have a noble work to perform, and their action in this organization gives token that they will perform it right nobly. The selection of Dr. A. S. Baldwin as their first President is an indication that the body was composed of working men, and that they mean *work*. Dr. Baldwin has stood upon the meteorological watch-tower there for *an age* already, and the world is indebted to his science, skill and untiring zeal and energy for much, not to say most, of what is now known of the climate and meteorology of Florida, as given through the Smithsonian Institute, and through other channels. He has in store a fund of knowledge that will add much to the medical history of his State, never yet given to the world, and we trust that through the medium of this association, over which he has been called to preside, if through none other, this knowledge will be cast as bread upon the waters, and that the fruits of his labors may stimulate others to emulate his example.

From the known character of those who have entered upon the work, we feel assured that this new field will, in due season, bring forth an abundant harvest. L.

From a private letter to the editor we learn that Dr. Calhoun, of Summerville, Chattooga county, Georgia, on the 25th January, delivered a lady in that county of a *trio of boys*, of whom two were living four days subsequently, and promised to do well. We hope Dr. Calhoun will send us a report of the case.

---

An unknown friend has favored us with a copy of the *Knoxville Daily Press and Herald*, in which we find a displayed double-column advertisement headed "A Card to the Afflicted," and elsewhere the advertising, itinerant doctor publishes two complimentary letters from personal and professional friends of ours, one now deceased—which, we feel the greatest assurance, would never have been given had the writers dreamed that such use was to be made of them in the public prints.

We notice this publication only to point the remark that physicians would do well to be very watchful in given written testimonials which could, by any chance, be thus used to their own mortification and the scandal of the brotherhood.

